# Inspiratory muscle weakness contributes to exertional dyspnea in chronic thromboembolic pulmonary hypertension

**DOI:** 10.1371/journal.pone.0204072

**Published:** 2018-09-27

**Authors:** João Victor Rolim, Jaquelina Sonoe Ota-Arakaki, Eloara V. M. Ferreira, Gabriela A. M. Figliolino, Ivan Ivanaga, Elaine Brito Vieira, Angelo X. C. Fonseca, Carolina M. S. Messina, Camila Melo Costa, J. Alberto Neder, Luiz Eduardo Nery, Roberta Pulcheri Ramos

**Affiliations:** 1 Pulmonary Circulation Group and Pulmonary Function and Exercise Physiology Unit, Division of Respiratory Diseases, Department of Medicine, Universidade Federal de São Paulo (Unifesp), São Paulo, SP, Brazil; 2 Laboratory of Clinical Exercise Physiology, Respiratory and Critical Care Division, Department of Medicine, Queen’s University, Kingston, ON, Canada; Ospedale del Cuore G Pasquinucci Fondazione Toscana Gabriele Monasterio di Massa, ITALY

## Abstract

Determination of potentially-reversible factors contributing to exertional dyspnea remains an unmet clinical need in chronic thromboembolic pulmonary hypertension (CTEPH). Therefore, we aimed to evaluate the influence of inspiratory muscle weakness (IMW) on exercise capacity and dyspnea during effort in patients with CTEPH. We performed a prospective cross-sectional study that included thirty-nine consecutive patients with CTEPH (48 ± 15 yrs, 61% female) confirmed by right heart catheterization that underwent an incremental cardiopulmonary exercise test, 6-minute walk test and maximum inspiratory pressure (MIP) measurement. MIP < 70%_pred_ was found in 46% of patients. On a multiple linear regression analysis, MIP was independently associated with 6MWD and $\dot{V}O_{2\;\mathrm{PEAK}}$. Patients with MIP < 70% presented greater $\Delta \dot{V}E/\Delta \dot{V}{\mathrm{CO}}_{2}$ than those with MIP ≥ 70%. Additionally, they also presented stronger sensations of dyspnea throughout exercise, even when adjusted for ventilation. At rest and at different levels of exercise, mean inspiratory flow (V_T_/T_I_) was significantly higher in patients with MIP < 70%. In conclusion, IMW is associated with a rapid increase of dyspnea, higher inspiratory load and poor exercise capacity in patients with CTEPH.

## Introduction

Chronic thromboembolic pulmonary hypertension (CTEPH) stands out as the only cause of pulmonary hypertension (PH) that can potentially be cured or ameliorated by pulmonary endarterectomy (PEA)[1]. Indeed, patients may show significant clinical improvement[2], better hemodynamics[3], and increased survival[4] after the procedure. However, 30–42% of patients are not considered for surgery[5–7], and despite advances in medical and percutaneous treatment[8], the prognosis is poor in nonsurgical CTEPH[9, 10]. In addition, surgical patients may still present exercise impairment after PEA[11–14]. Therefore, identification of the contributing factors that may influence exercise capacity in CTEPH would be helpful for the development of new therapeutic strategies and prognostic assessment.

In this context, there is intriguing evidence that PH is associated with a well-known cause of exertional dyspnea: inspiratory muscle weakness[15–19]. Although the underlying reasons are not fully understood, it has been postulated that chronic diaphragmatic “overload” due to increased ventilation[20] in a patient suffering the negative trophic influences of chronic inflammation[21], oxidative-stress[22] and poor perfusion[23] would lead to an imbalance between protein synthesis and degradation with consequent dysfunction[19]. For instance, Manders et al. found that maximal force-generating capacity was reduced in diaphragm slow-twitch muscle fibers whereas the calcium sensitivity of force generation was reduced in fast-twitch muscle fibers of CTEPH patients compared to controls. Those micro-structural abnormalities were associated with reduced global respiratory muscle strength as shown by a low maximal “static” inspiratory pressure (MIP)[24].

Of note, CTEPH is associated with unordinary-high exercise $\dot{V}E$; for instance, CTEPH patients show the steepest $\Delta \dot{V}E‑\Delta {\mathrm{CO}}_{2}$ output ($\dot{V}{\mathrm{CO}}_{2}$) slope during incremental exercise among several cardio-respiratory diseases[25]. It follows that patients’ inspiratory muscles might be particularly prone to overloading and dysfunction. Thus, inspiratory muscle weakness, in addition to excessive ventilation, might constitute a hitherto unexplored factor associated with exertional dyspnoea in CTEPH.

## Patients and methods

### Study design

After ethics approval (Medical Ethics Committee, Universidade Federal de São Paulo), we conducted a single center prospective cross-sectional study in which thirty-nine consecutive patients with an established diagnosis of CTEPH[26] were prospectively selected between 2014–2016. Written informed consent was obtained from all patients.

### Study sample

Inclusion criteria were patients aged 18 to 75 years, right heart catheterization performed up to 6 months prior to functional evaluation and showing mean pulmonary artery pressure (mPAP) ≥ 25 mmHg and occlusion pressure of pulmonary artery (PAOP) ≤ 15mmHg at rest, New York Heart Association (NYHA) functional class II to IV without use of continuous home oxygen therapy and clinical stability in the last 2 months (no change in therapy, no worsening of symptoms, absence of syncope, no need for hospitalization). All patients underwent lung scintigraphy and thoracic computed tomograghy at time of diagnosis and 59% presented proximal (main or lobar) lesions. Exclusion criteria were the presence of concomitant left-heart cardiovascular disease, other obstructive, interstitial or sleep-related pulmonary disorders or associated connective tissue diseases. Informed consent was obtained from each patient.

### Measurements

#### Pulmonary function tests

Spirometric measurements were performed using Clinical Pulmonary Function—Spirometry System (CPF-S, Medical Graphics Corporation, St. Paul, MN, USA). The patients completed at least three forced expiratory maneuvers acceptable and reproducible according to established criteria. Variables obtained were forced expiratory volume in 1s (FEV_1_); forced vital capacity (FVC) and maximal voluntary ventilation (MVV)[27, 28]. In addition, 31 patients were able to perform diffusion capacity for carbon monoxide maneuvers.

#### Cardiopulmonary exercise test (CPET)

All patients performed a ramp incremental CPET (5-10W/min) limited by symptoms on a cycle ergometer of lower limbs (ULTIMA CPX Medical Graphics Corporation). All tests were preceded by two minutes of warm-up period. Patients were asked about the feeling of breathlessness and fatigue of the lower limbs by the modified Borg scale every 3 minutes. The following variables were obtained breath-by-breath during the CPET: oxygen uptake ($\dot{V}O_{2}$, mL/min), carbon dioxide output ($\dot{V}{\mathrm{CO}}_{2}$, mL/min), respiratory exchange ratio (R), minute ventilation ($\dot{V}E$, L/min), respiratory rate (*f*, rpm), tidal volume (V_T_, L), ventilatory equivalent for O_2_ and CO_2_ ($\dot{V}E/\dot{V}O_{2}$ and $\Delta \dot{V}E/\dot{V}{\mathrm{CO}}_{2}$) and partial end-expiratory pressures of O_2_ and CO_2_ (P_ET_O_2_ and P_ET_CO_2_, mmHg). A twelve-lead eletrocardiogram was continuously analyzed, as well as oxyhemoglobin saturation by pulse oximetry (SpO_2_). $\dot{V}O_{2\;\mathrm{PEAK}}$ was the average value during the last 15 sec of the ramp and was compared with a reference equation[29]. $\dot{V}O_{2}$ at the gas exchange threshold (GET) was estimated by gas exchange and ventilatory methods. Sub-maximal relationships were established as described elsewhere[30]. Specifically, $\Delta \dot{V}E/\Delta \dot{V}{\mathrm{CO}}_{2}$ was calculated from the start of the work rate increment to the respiratory compensation point (RCP).

#### Six-minute walk test (6MWT)

The 6MWT was performed as ATS/ERS guidelines, using a corridor of 30 meters[31]. The parameters evaluated were walked distance (6MWD), SpO_2_, heart rate (HR) and the modified Borg scale at the end of test.

#### Respiratory muscle strength

Evaluation of respiratory muscle strength was measured by an analog manovacuometer (Farmabas®, Sao Paulo, Br). Measurement of MIP was performed from the residual volume, in accordance with the recommendations of the ATS/ERS[32]. The maximum value of three maneuvers that varied by less than 10% was recorded. We *a priori* established that values < 70% predicted by Neder et al.’s equations[28] were indicative of low MIP. This threshold was defined based on the results of Rodrigues et al. showing that MIP < 70% according to this specific frame of reference was associated with a cluster of clinical and functional findings consistent with inspiratory muscle weakness[33].

### Statistical analysis

A statistical software was used for data analysis (SPSS, version 19.0, Chicago, IL, USA). Results are summarized as mean ± SD or median [25–75% interquartile range]. Differences between patients with MIP < 70% and MIP ≥ 70% were compared by using a non-paired *t* test or Mann-Whitney test. A *p* value less than 0.05 was considered statistically significant. Comparison of data over exercise time points was performed by Repeated measures ANOVA. Multivariable analysis was performed to adjust MIP and exercise capacity association for clinical (NYHA functional class) and haemodynamic (pulmonary vascular resistance) confounders. Sample size was calculated considering previous studies showing a mean VO_2PEAK_ of 12 ± 3mL/Kg/min in patients with CTEPH. Therefore, to detect a minimum difference of 3mL/Kg/min, α(two-sided) = 0.05 and β = 0.10, 18 patients are required per group. Similar results were obtained considering a difference of 2 points on Borg dyspnea scale.

## Results

### General characteristics

We found that 18/39 (46%) patients presented with MIP < 70%. These patients showed lower forced vital capacity (and, proportionally, FEV_1_) and maximal voluntary ventilation compared to their counterparts with MIP ≥ 70% (Table 1).

**Table 1:** Baseline characteristics of patients with chronic thromboembolic pulmonary hypertension showing maximum inspiratory pressure < or ≥ 70% predicted.

	MIP < 70% (n = 18)	MIP ≥ 70% (n = 21)	*p* value
***Demographics / Anthropometric***			
Gender F [n (%)]	12 (66)	12 (57)	0.39
Age (years)	49 ± 17	48 ± 14	0.93
BMI (kg/m^2^)	26.3 ± 5.6	29.6 ± 4.2	0.04
***Surgical CTEPH* [n (%)]**	10 (55)	13 (62)	0.55
***NYHA functional class* [n (%)]**			
II	6 (33)	14 (67)	0.039
III/IV	12 (67)	7 (33)	
***Medications***			
Sildenafil [n/(%)]	2 (11)	1 (5)	
Bosentan [n/(%)	3 (17)	3 (14)	
Riociguat [n/(%)]	0	3 (14)	
Combination	3(17)	0	
***Hemodynamics***			
mPAP (mmHg)	55 ± 12	50 ± 14	0.23
PVR (*dynes*.s.cm^-5^)	908 ± 300	799 ± 402	0.35
RAP (mmHg)	13 ± 4	11 ± 4	0.18
Cardiac index (L/min/m^2^)	2.3 ± 0.6	2.4 ± 0.8	0.40
***Blood gas analysis***			
PaO_2_ (mmHg)	62 ± 9	64 ± 11	0.689
PaCO_2_ (mmHg)	30 ± 5	31 ± 4	0.762
SaO_2_ (mmHg)	91 ± 3	92 ± 3	0.771
***Pulmonary function tests***			
MVV (L)	92 ± 26	111 ± 22	0.013
FEV_1_ (% pred)	71 ± 12	83 ± 12	0.003
FVC (% pred)	76 ± 11	87 ± 13	0.005
FEV_1_/FVC	0.76 ± 0.07	0.73 ± 0.17	0.49
D_L_CO (% pred)	59 ± 17	64 ± 15	0.41

Data are expressed as the mean ± standard deviation or *n* (%), as indicated. F: female; BMI: body mass index; NYHA: New York Heart Association; mPAP: mean pulmonary artery pressure; RAP: right atrial pressure; PVR: pulmonary vascular resistance; PaO_2_: arterial partial pressure of oxygen; PaCO_2_: arterial partial pressure of carbon dioxide; SaO_2_: arterial oxyhemoglobin saturation; MVV: maximum voluntary ventilation; FEV_1_: forced expiratory volume in 1s; FVC: forced vital capacity; D_L_CO: diffusion capacity for carbon monoxide (n = 31).

Despite similar cardiac index and mPAP (p>0.05), the former group also reported lower functional capacity in daily life. Thus, whereas 12/18 (67%) patients from the lower MIP group were on NYHA class 3–4, 14/21 (67%) from the higher MIP group were on NYHA class 2. This was confirmed by lower 6-min walking distance and peak work rate (Table 2). There were no significant between-group differences in exertional O_2_ desaturation (p>0.05).

**Table 2:** Six-minute walk test and cardiopulmonary exercise test responses in patients with chronic thromboembolic pulmonary hypertension showing maximum inspiratory pressure < or ≥ 70% predicted.

	MIP < 70% (n = 18)	MIP ≥ 70% (n = 21)	p value
***Six-minute walk test***			
6MWD (m)	352 ± 116	438 ± 68	0.007
6MWD (% pred)	65 ± 20	78 ± 11	0.013
SpO_2_ (%)	85 ± 8	87 ± 5	0.450
***Cardiopulmonary exercise test***			
WR _PEAK_ (watts)	47 ± 23	78 ± 27	0.001
$\dot{V}O_{2\;\mathrm{PEAK}}$ (mL/Kg/min)	10.5 ± 2.4	13.1 ± 3.0	0.005
$\dot{V}O_{2\;\mathrm{PEAK}}$ (% pred)	45.7 ± 11.1	63.9 ± 18.7	0.001
$\dot{V}O_{2\;\mathrm{GET}}$ (mL/min)‡	494 ± 127	689 ± 157	0.002
$\Delta \dot{V}O_{2}/\mathrm{\Delta W}$	7 ± 3	9 ± 2	0.102
$\dot{V}O_{2}/{\mathrm{HR}}_{\mathrm{PEAK}}$ (mL/beat)	5.7 ± 1.4	7.4 ± 1.8	0.002
$\Delta \mathrm{HR/}\Delta \dot{V}O_{2}$	98 ± 42	91 ± 31	0.573
$\Delta \dot{V}E/\Delta \dot{V}{\mathrm{CO}}_{2}$	64 ± 12	50 ± 8	<0.001
V_T REST_ (L)	0.57 ± 0.16	0.70 ± 0.29	0.089
V_T PEAK_ (L)	1.37 ± 0.44	1.72 ± 0.37	0.011
$\dot{V}E/\mathrm{MVV}$	0.57 ± 0.14	0.60 ± 0.09	0.39

Data are expressed as the mean ± standard deviation. Abbreviatures: 6MWD: six-minute walk distance; SpO_2_: oxyhemoglobin saturation by pulse oximetry; WR: work rate; $\dot{V}O_{2}$: oxygen uptake; GET: gas exchange threshold; HR: heart rate; $\dot{V}E$: minute ventilation; $\dot{V}{\mathrm{CO}}_{2}$: carbon dioxide output; V_T_: tidal volume; MVV: maximal voluntary ventilation.

‡ Evaluated in 29 patients.

### Association with exercise capacity

**Fig 1:**
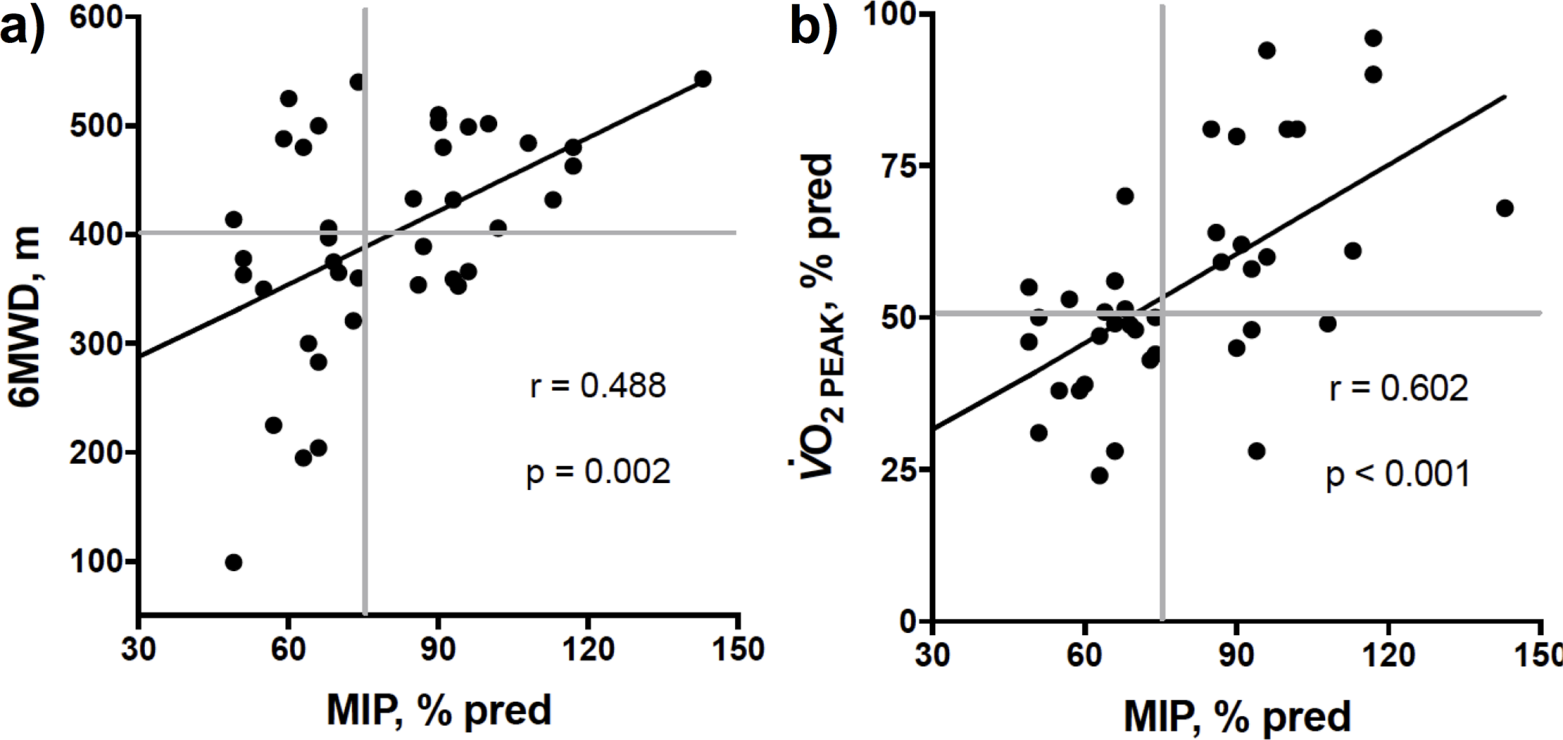
A) Six-minute walking distance (6MWD) and B) oxygen consumption at the peak of cardiopulmonary exercise test ($\dot{V}O_{2\;\mathrm{PEAK}}$) as a function of maximum inspiratory pressure (MIP). Horizontal gray lines represent median of variables.

MIP was positively correlated with both 6MWD and $\dot{V}O_{2\;\mathrm{PEAK}}$ (Fig 1). On a multiple linear regression analysis, including pulmonary vascular resistance (PVR) and New York Heart Association functional class (NYHA FC), MIP was independently associated with $\dot{V}O_{2\;\mathrm{PEAK}}$ (partial r 0.480, p 0.003). Similar results were found for 6MWD as outcome variable (partial r 0.361, p 0.028).

### Ventilatory responses and dyspnea sensation

**Fig 2:**
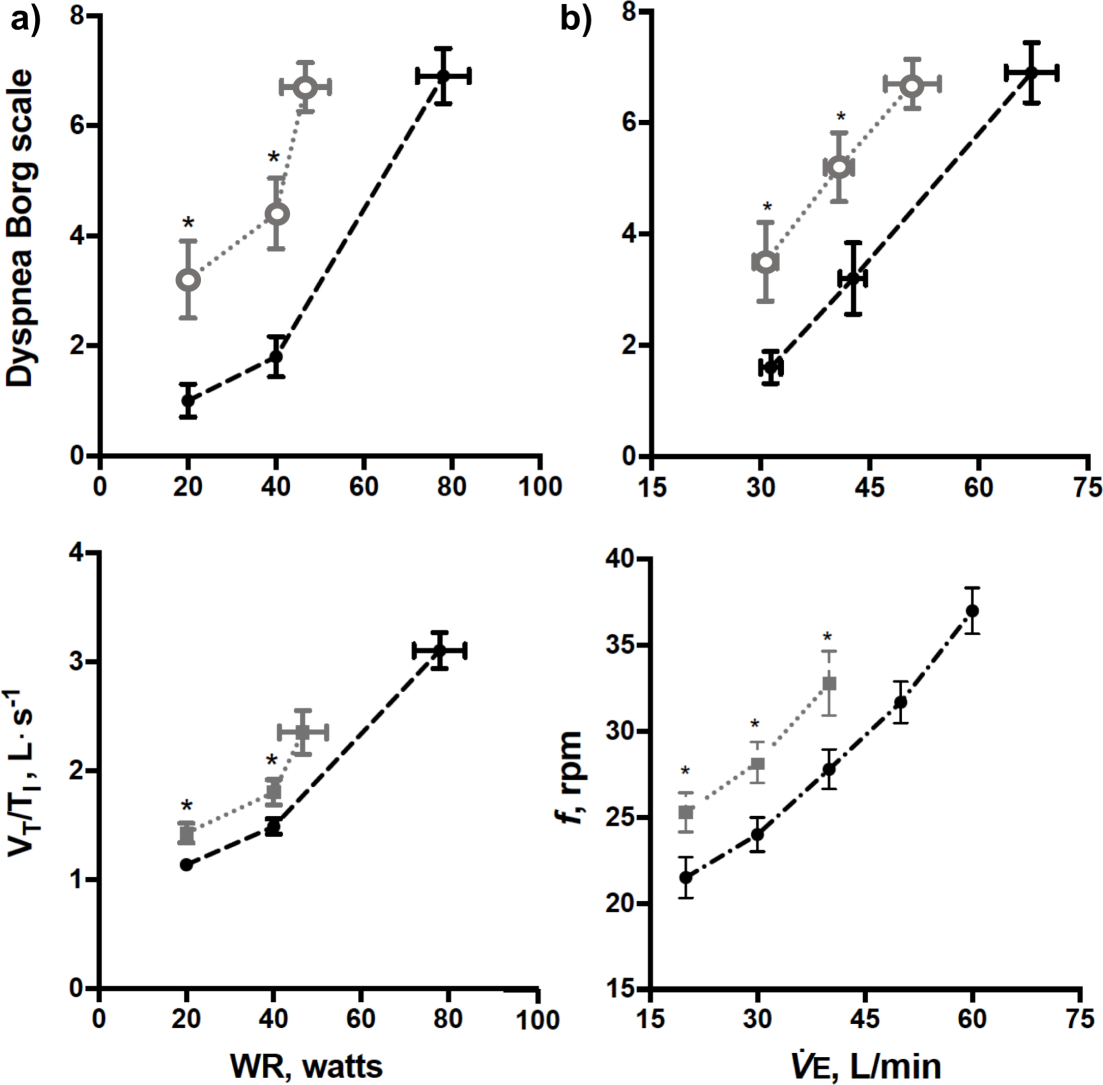
**Borg dyspnea ratings as a function of work rate (WR) (*panel A*) and minute ventilation (**$\dot{V}E$**) (*panel B*) in CTEPH patients showing maximal inspiratory pressure (MIP) < or ≥ 70% predicted according to Neder et al.’s reference equations (*closed* and *open* symbols, respectively)**. *Panel C* and *D* shows tidal volume (V_T_) /inspiratory time (T_I_) ratio–a crude index of neural drive–and respiratory frequency (*f*) as a function of $\dot{V}E$ (*p < 0.05).

The MIP < 70% group reported higher dyspnoea ratings for a given work rate (Fig 2, *panel A*) and $\dot{V}E$ (Fig 2, *panel A*). These findings were also associated with higher mean inspiratory flow (Fig 2, *panel C*) and faster respiratory rate (Fig 2, *panel D*) at iso-work rate and $\mathrm{iso}‑\dot{V}E$. Moreover, these patients had lower exertional tidal volume (VT) (1.37 ± 0.44 L *vs*. 1.72 ± 0.37 L at peak exercise; p<0.05). In line with our hypothesis, there was a significant negative relationship between MIP and $\Delta \dot{V}E/\Delta \dot{V}{\mathrm{CO}}_{2}$ (r = -0.49, p<0.05). Particularly high end-exercise dyspnea scores were found in 9 patients in whom MIP < 70% was associated with severely increased $\Delta \dot{V}E/\Delta \dot{V}{\mathrm{CO}}_{2}$ (>60) (median (interquartile range) = 7 (3)); conversely, lower dyspnea burden was found in 7 patients with MIP ≥ 70% and $\Delta \dot{V}E/\Delta \dot{V}{\mathrm{CO}}_{2}$ < 50 (4 (2.5)). In those with intermediate $\Delta \dot{V}E/\Delta \dot{V}{\mathrm{CO}}_{2}$ (50–60), dyspnea was higher at $\mathrm{iso}-\dot{V}E$ in those with MIP < 70% (N = 8) compared with their counterparts (p<0.05).

## Discussion

This is the first study to investigate the association of low MIP with exertional breathlessness of patients with CTEPH. We demonstrated that patients with MIP < 70% presented worse exercise capacity and a rapid increase of dyspnea sensation during exercise, compared to those with MIP ≥ 70%.

Recent research evaluated diaphragm muscle fiber contractility and inspiratory muscle function in 13 patients with surgical CTEPH^24^. Their main result was that patients presented reduced maximal tension and cross-sectional area of the slow-twitch fibers, as well as reduced heavy-chain myosin concentration and calcium sensitivity of the fast-twitch fibers in comparison to a healthy control group. Our study adds to the current knowledge by including a detailed physiopathological analysis of the association of reduced MIP with ventilatory responses and dyspnea during exercise.

Our cross-sectional, observational study precludes strong mechanistic insights into the genesis of low MIP in CTEPH patients. Insufficient O_2_ delivery relative to increased O_2_ needs in patients with highest exertional $\dot{V}E$ might have contributed to decreased inspiratory muscle strength, particularly in patients with $\Delta \dot{V}E/\Delta \dot{V}{\mathrm{CO}}_{2}$ > 60. Indeed, it has long been recognized that O_2_ requirements of the respiratory muscles increase exponentially with $\dot{V}E$ [34]; thus, it is conceivable that under constrained cardiac output, tissue hypoxia develop even at relatively mild physical demands. Previous studies demonstrating absence of peripheral muscle weakness in patients with low MIP[19] argues against systemic myopathy. Due to patients’ immobility, however, the appendicular muscles might be relatively spared from the ominous consequences of chronic hypoxia under high contractile regimens. Thus, any negative systemic influence (e.g., low-grade inflammation[35], hyper-oxidative stress[22, 36]) might be particularly deleterious to the overloaded respiratory muscles. Sympathetic over-excitation[37] is another potential mechanism underlying both high $\Delta \dot{V}E/\Delta \dot{V}{\mathrm{CO}}_{2}$ and impaired diaphragmatic perfusion leading to poor oxygenation. Considering low MIP at iso-$\Delta \dot{V}E/\Delta \dot{V}{\mathrm{CO}}_{2}$ in some patients with intermediate $\Delta \dot{V}E/\Delta \dot{V}{\mathrm{CO}}_{2}$, other hitherto unknown mechanisms are likely to be involved in CTEPH-related inspiratory muscle weakness.

Some of our findings provide useful insights into the neurophysiology of dyspnea in CTEPH. For instance, increased $\Delta \dot{V}E/\Delta \dot{V}{\mathrm{CO}}_{2}$ in response to high respiratory neural drive is a well-established cause of high dyspnea for a given work rate[38]. If drive is fully translated into lung-chest wall displacement, dyspnea tends to increase in tandem with the system output. i.e., $\dot{V}E$ [39]. On other hand, when impaired lung mechanics (including respiratory muscle weakness) compound with high neural drive, dyspnea is expected to increase at a given work rate *and* $\dot{V}E$ [40]. Under these circumstances, a high system input (respiratory drive) cannot be readily translated into an equally high output ($\dot{V}E$). In fact, even if this proves partially feasible, it implies into additional muscle fibers recruitment–which is likely to further increase neural drive in a vicious circle. In the present study, low MIP was associated with higher dyspnea for a given $\dot{V}E$ throughout exercise. This finding is consistent with the interpretation that part of the heightened respiratory neural drive was not fully translated into (even higher) $\dot{V}E$ due to inspiratory muscle weakness.

The association between high $\Delta \dot{V}E/\Delta \dot{V}{\mathrm{CO}}_{2}$ and low MIP was particularly noticeable. For instance, patients with higher dead space (VD) characteristically present with higher $\dot{V}E$ which, as discussed, may predispose to respiratory muscle dysfunction due to poor local O_2_ delivery. On the other hand, lower VT due to inspiratory muscle weakness is expected to increase VD/VT ratio, a strong determinant of $\Delta \dot{V}E/\Delta \dot{V}{\mathrm{CO}}_{2}$ in CTEPH. In fact, most patients with higher $\Delta \dot{V}E/\Delta \dot{V}{\mathrm{CO}}_{2}$ (>60) presented with low MIP; conversely, the majority of patients with lower $\Delta \dot{V}E/\Delta \dot{V}{\mathrm{CO}}_{2}$ (<50) had preserved MIP. Thus, it could be argued that $\Delta \dot{V}E/\Delta \dot{V}{\mathrm{CO}}_{2}$ was “the” primary mechanism behind patients’ dyspnea. However, the group with intermediate $\Delta \dot{V}E/\Delta \dot{V}{\mathrm{CO}}_{2}$ showed a large range of MIP values (45–140%) thereby allowing comparison of low versus preserved MIP at similar $\Delta \dot{V}E/\Delta \dot{V}{\mathrm{CO}}_{2}$. This analysis showed that the former group has worse dyspnea at a given $\dot{V}E$ – which further corroborates the notion that inspiratory muscle weakness contributed to exertional dyspnea.

What are the implications of our results? In clinical practice, respirologists are frequently faced with CTEPH patients presenting with “out-of-proportion” (to echocardiographic and hemodynamic data) shortness of breath. Our results indicate that referring these patients to pulmonary function testing for MIP measurement and incremental CPET might prove valuable to clarify the underlying reasons. Identifying patients with low MIP and high $\Delta \dot{V}E/\Delta \dot{V}{\mathrm{CO}}_{2}$ might impact on clinical-decision making. Indeed, our results may provide important insights into the pathophysiological mechanisms of exercise intolerance in patients with CTEPH. These findings could contribute considerably to the development of inspiratory muscle training protocols as an adjunctive therapy for such patients. If improvement in MIP with inspiratory muscle training translates into low exertional dyspnea and better functional capacity, this non-pharmacological intervention might be clinically valuable in selected patients.

Some study limitations open novel perspectives for clinical physiology research applied to CTEPH. Measurements of operating lung volumes using serial inspiratory capacity maneuvers during exercise might provide important clues on whether higher dyspnoea scores are associated with critical inspiratory constraints. Due to high prevalence of inspiratory muscle weakness in CTEPH, it seems advisable that esophageal pressures are concomitantly measured to better interpret potential changes in inspiratory capacity. MIP is a crude index of global (diaphragm and accessory respiratory muscles) inspiratory muscle strength being highly effort dependent. Thus, studies using more advanced, non-volitional techniques are clearly warranted to confirm that low MIP is indeed an index of diaphragmatic weakness, e.g., phrenic nerve stimulation and diaphragm electromyography. Ultrasound evaluation of the diaphragm is also a simple and non-invasive test for use both in the clinical and research settings [41]. In practice, however, static respiratory muscle pressures remain the most common measurements to screen for weakness. Our findings indicating that 70% MIP as predicted by Neder et al.’s equations discriminate CTEPH patients with worse functional capacity, poorer exercise tolerance and higher dyspnea burden provides support for this threshold to suggest clinically-relevant weakness. Care should be taken, however, that due to the large differences in predicted MIP according to different frames of reference, the 70% cut-off is unlikely to be applicable to all prediction equations.

We reinforce that the lack of non-volitional strength tests precludes any inference regarding diagnostic precision. However, advanced tests are not available in most non-specialized pulmonary function laboratories. Finally, MIP is a low cost and easy to perform with a portable equipment that may be used to identify CTEPH patients with disproportional dyspnea during effort.

## Conclusion

In conclusion, low MIP is commonly seen in CTEPH, being associated with worse activity-related shortness of breath and functional capacity. As cogently pointed out by Naeije, " breathing more with weaker respiratory muscles"[42] in pulmonary vascular disease represents an unfortunate combination with negative sensory effects (high dyspnea burden) leading to ominous clinical consequences (poor tolerance to exertion).
